# Estimating the burden of influenza-attributable severe acute respiratory infections on the hospital system in Metropolitan France, 2012–2018

**DOI:** 10.1186/s12879-023-08078-2

**Published:** 2023-03-06

**Authors:** Anne Bernadou, C. Sommen, M. Pivette, B. Hubert

**Affiliations:** 1 Santé publique France, the national public health agency – Nouvelle-Aquitaine, Bordeaux, France2 France; 2 Santé publique France, the national public health agency, Saint-Maurice, France; 3 Santé publique France, the national public health agency – Bretagne, Rennes, France

**Keywords:** Influenza, Severe acute respiratory infections (SARI), Surveillance, Hospital, Burden

## Abstract

**Background:**

To date, estimating the burden of seasonal influenza on the hospital system in France has been restricted to influenza diagnoses in patients (estimated hospitalization rate of 35/100,000 on average from 2012 to 2018). However, many hospitalizations for diagnosed respiratory infections (e.g. pneumonia, acute bronchitis) occur without concurrent screening for virological influenza, especially in the elderly. Specifically, we aimed to estimate the burden of influenza on the French hospital system by examining the proportion of severe acute respiratory infections (SARI) attributable to influenza.

**Methods:**

Using French national hospital discharge data from 1/7/2012 to 30/6/2018, we extracted SARI hospitalizations with ICD-10 codes J09-J11 (influenza codes) in main or associated diagnoses, and J12-J20 (pneumonia and bronchitis codes) in main diagnoses. We estimated influenza-attributable SARI hospitalizations during influenza epidemics, as the number of influenza-coded hospitalizations plus the influenza-attributable number of pneumonia- and acute bronchitis-coded hospitalizations using periodic regression and generalized linear models. Additional analyses stratified by age group, diagnostic category (pneumonia and bronchitis), and region of hospitalization were performed using the periodic regression model only.

**Results:**

The average estimated hospitalization rate of influenza-attributable SARI during the five annual influenza epidemics covered (2013–2014 to 2017–2018) was 60/100,000 with the periodic regression model, and 64/100,000 with the generalized linear model. Over the six epidemics (2012–2013 to 2017–2018), of the 533,456 SARI hospitalizations identified, an estimated 227,154 were influenza-attributable (43%). Fifty-six percent of cases were diagnosed with influenza, 33% pneumonia, and 11% bronchitis. Diagnoses varied between age groups: 11% of patients under 15 years old had pneumonia versus 41% of patients aged 65 and older.

**Conclusion:**

Compared to influenza surveillance in France to date, analyzing excess SARI hospitalizations provided a much larger estimate of the burden of influenza on the hospital system. This approach was more representative and allowed the burden to be assessed according to age group and region. The emergence of SARS-Cov-2 has led to a change in the dynamics of winter respiratory epidemics. The co-circulation of the three current major respiratory viruses (influenza, SARS-Cov-2, and RSV) and the evolution of diagnostic confirmation practices must now be taken into account when analyzing SARI.

**Supplementary Information:**

The online version contains supplementary material available at 10.1186/s12879-023-08078-2.

## Background

France’s influenza surveillance system, led by Santé Publique France, the national public health agency, is based on several sources of inpatient and outpatient data [1]. The severity and impact of seasonal influenza on the health system are routinely monitored using two indicators: the number of patients visiting emergency departments (ED) who are subsequently hospitalized, and cases admitted to intensive care units (ICU). Although these two indicators allow real-time monitoring during the influenza season, they only reflect part of the burden of influenza on the hospital system. This is because of the data sources used, and a definition limited to clinical influenza diagnosis [2].

The French national hospital discharge database (PMSI) records information on all patients hospitalized a posteriori. Two French studies have recently used this database to estimate the epidemiological and economic burdens of severe influenza in the country [3] and to describe the characteristics and severity of patients hospitalized with diagnosed influenza [2]. However, many hospitalizations for respiratory infections (e.g., pneumonia, bronchitis) occur without virological influenza concurrently diagnosed [4].

In order to compensate for this lack of virological confirmation for atypical forms of influenza or forms with complications, the World Health Organization (WHO) has proposed broadening influenza surveillance by monitoring severe acute respiratory infections (SARI) [5]. This provides a more representative assessment of the true burden of influenza epidemics on the hospital system.

We aimed to estimate the burden of influenza-attributable SARI on the hospital system in metropolitan France over six annual epidemics (2012 to 2018) using two regression models, specifically a periodic regression model and a generalized linear model (GLM). The purpose of the study was to generate a robust indicator that is more representative of the true burden of winter epidemics of respiratory infections attributable to influenza on the hospital system.

## Methods

### Hospitalization data

The PMSI database is part of France’s larger National Health Data System (Système national des données de santé, SNDS) database. From the PMSI, we extracted all hospitalizations with a diagnosis of SARI (ICD-10 code: J09-J20) occurring between 1 July 2012 and 30 June 2018. For each hospitalization, we obtained the following information: age group, week of admission, total length of stay (in days), admission to an intensive care unit (yes/no), and death (yes/no).

### Definitions

Based on the definition of SARI proposed by WHO [5] and consistent with the surveillance performed in France, the case definition of SARI was defined as any hospitalization with (i) a diagnosis of influenza as the principal (PD) or associated diagnosis (AD), or (ii) a diagnosis of pneumonia as the principal diagnosis, or (iii) a diagnosis of bronchitis as the principal diagnosis.

Influenza epidemic periods from the 2012 to 2018 seasons were identified from nationally calculated thresholds using three outbreak detection methods applied to three surveillance data sources: emergency departments, emergency general practitioners, and sentinel general practitioners (Additional file 1) [6]. SARI were categorized into three diagnostic categories: influenza (influenza PD or influenza AD associated with a PD other than bronchitis or pneumonia), pneumonia (pneumonia PD with or without influenza AD), and acute bronchitis (bronchitis PD with or without influenza AD). Five age-groups were defined: <15 years, 15–64 years, 65–74 years, 75–84 years, and > = 85 years.

### Statistical analysis

The weekly time series of hospitalizations for pneumonia and bronchitis were analyzed with statistical methods. We briefly describe the methods used below. All statistical analyses were performed with R-3.5.1.

#### Periodic regression

To estimate the proportion of influenza-attributable SARI, a periodic regression method was applied to the weekly number of hospitalizations for pneumonia and bronchitis [7]. We modelled the baseline level with a multivariable linear regression model using the following equation:

$$\begin{array}{l}E\left( {{y_w}} \right) = {\alpha _0} + {\alpha _1}w + {\gamma _1}cos\left( {2\pi w/52.17} \right)\\\,\,\,\,\,\,\,\,\,\,\,\,\,\,\,\, + {\delta _1}sin\left( {2\pi w/52.17} \right) + {\gamma _2}cos\left( {4\pi w/52.17} \right)\\\,\,\,\,\,\,\,\,\,\,\,\,\,\,\,\, + {\delta _2}sin\left( {4\pi w/52.17} \right),\end{array}$$

where y_w_ is, for week w, the number of pneumonia and bronchitis hospitalizations.

The baseline level in a given week w was obtained by fitting the model to the observations from 1/07/2012 to 30/06/2018 from which we removed the presence of influenza and bronchiolitis epidemics (Additional file 1) in the training dataset from the series. This trimming was an attempt to ensure that the dataset used to model the baseline level was free of influenza or respiratory syncytial virus (RSV) [8].

The number of excess hospitalizations for pneumonia or bronchitis was defined as the sum of the differences between the expected and observed values. This excess was considered attributable to influenza during periods defined as influenza epidemics.

#### Generalized linear model

We also performed a generalized linear model (GLM) as a complementary approach to validate the results of the periodic regression method. Specifically, the GLM was performed to estimate the number of influenza-associated hospitalizations during the epidemic periods by using overall pneumonia and bronchitis (PD) hospitalizations, assuming negative-binomial distributed errors with a logarithmic link function.

Two model parameters were used: indicators of influenza activity (including influenza-like illness (ILI) incidence data and virological data), and morbidity data for RSV. Weekly ILI incidence data were obtained from the French general practitioner network Sentinelles. Percentages of nasopharyngeal samples testing positive for influenza in France were obtained from the National Reference Center for Influenza. Swabs were performed by practitioners in the Regional Groups for Influenza Surveillance Network (GROG) (until the 2013/2014 influenza season) and by members of the Sentinelles network (from the 2014/2015 influenza season onward). These data were stratified by influenza type and subtype: A(H1N1)pdm09, A(H3N2) and B. For each influenza type and subtype, the product of ILI incidence and the percentage of samples testing positive was used as an indicator of influenza activity. As a proxy for the circulation of RSV, the proportions of consultations for bronchiolitis were obtained from computerized medical records completed during consultations at emergency departments participating in the OSCOUR® network (representing from approximatively 50% of national emergency department activity in 2010-11 to 90% in 2016-17). Model parameters are described in Additional file 2.

The GLM model analysis could only be performed on the five seasons from 2013 to 14 to 2017-18 because of the lack of availability of virological data for influenza before 2013.

The model was adjusted for temporal trend and RSV circulation. We selected lags on influenza activity indicators (lags retained: 0, 1) and the RSV indicator (lags 0, 1), based on the Akaike Information Criterion (AIC). In order to take into account the nonlinear relationship between hospitalizations for pneumonia and bronchitis and covariables, we used b-splines (with three degrees of freedom) on each component [9]. Population figures were introduced into the model as an offset.

The following regression model was used:

$$\begin{array}{l}{\rm{log}}(E({Y_t}/Pop)) = {\beta _0} + {\beta _1}bs\left( t \right)\\\,\,\,\,\,\,\,\,\,\,\,\,\,\,\,\,\,\,\,\,\,\,\,\,\,\,\,\,\,\,\,\,\,\,\,\, + {\beta _8}bs(flag(IL{I_{H1N1}}_t))\\\,\,\,\,\,\,\,\,\,\,\,\,\,\,\,\,\,\,\,\,\,\,\,\,\,\,\,\,\,\,\,\,\,\,\,\, + {\beta _9}bs(flag(IL{I_{H3N2}}_t))\\\,\,\,\,\,\,\,\,\,\,\,\,\,\,\,\,\,\,\,\,\,\,\,\,\,\,\,\,\,\,\,\,\,\,\,\, + {\beta _{10}}bs(flag(IL{I_B}_t))\\\,\,\,\,\,\,\,\,\,\,\,\,\,\,\,\,\,\,\,\,\,\,\,\,\,\,\,\,\,\,\,\,\,\,\,\, + {\beta _{11}}bs(flag(Bronchi{o_t}))\end{array}$$

where ${Y}_{t}$ was the number of pneumonia and bronchitis hospitalizations predicted by the model, $Pop$ was the offset corresponding to the population size, $t$was the time, $bs$ a function of b-splines with three degrees of freedom, and $flag$ a function of lag.

The number of pneumonia and bronchitis hospitalizations attributable to influenza was estimated as the difference between the number predicted by the model and the number predicted by the model in the absence of influenza virus circulation. The numbers of weekly pneumonia and bronchitis hospitalizations attributable to influenza estimates were summed to obtain estimates during the five epidemic periods.

#### Estimated number of influenza-attributable SARI

The number of influenza-attributable SARI hospitalizations was estimated as the number of pneumonia and bronchitis hospitalizations attributable to influenza plus the number of influenza hospitalizations.

The proportion of influenza-attributable SARI hospitalizations was calculated by dividing the number of influenza-attributable SARI hospitalizations by the overall number of SARI hospitalizations during the six influenza epidemics for periodic regression model and five influenza epidemics for GLM.

Hospitalization rates for influenza-attributable SARI were then calculated from the estimated numbers of influenza-attributable SARI - according to each method applied - compared with population size estimates provided by the French National Institute of Statistics and Economics Studies (2016).

For each of the two methods used, confidence intervals (CI95%) of the hospitalization rate estimates were calculated for each influenza epidemic.

Using the periodic regression model, estimates were stratified by age-group, diagnostic category (pneumonia and bronchitis), and region of residence.

## Results

### Description of SARI hospitalizations

**Fig. 1:**
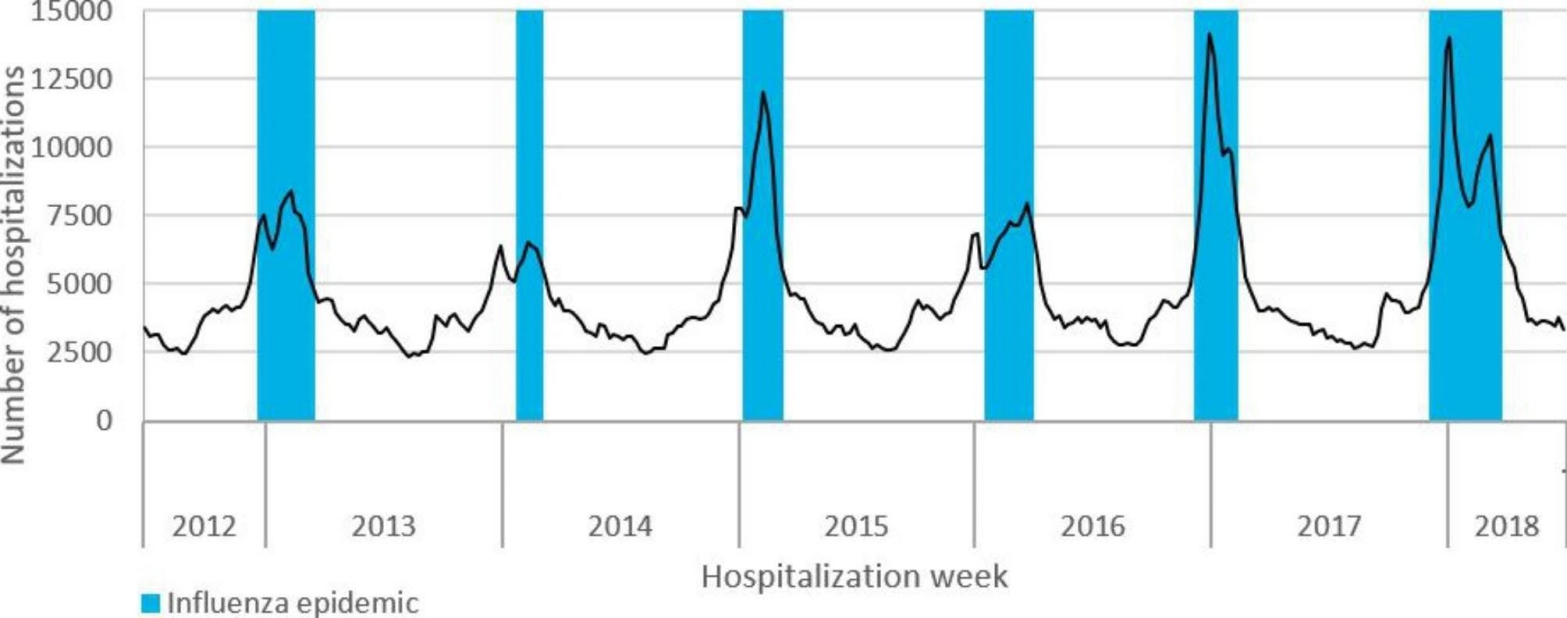
Weekly number of SARI hospitalizations and influenza season (in blue), 2012–2018, metropolitan France

From July 2012 to June 2018, 533 456 SARI hospitalizations were recorded during the six influenza epidemics in metropolitan France (Fig. 1). Over half concerned persons aged 75 years or older. The proportion of hospitalizations with ICU admission (6%), and the case fatality rate (6%) were stable over all six epidemics. Among all SARI, the proportion of hospitalizations with a diagnosis of influenza tended to increase over all epidemic seasons from 14 to 31% (Table 1). Among hospitalizations with a diagnosis of influenza, 65% had influenza as a PD and 35% as an AD.

**Table 1:** Description of SARI hospitalizations for each influenza epidemic, 2012–2018, metropolitan France

	2012–2013	2013–2014	2014–2015	2015–2016	2016–2017	2017–2018	Total
Epidemic duration (in wk)^a^	13	6	9	11	10	16	/
Number of SARI hospitalizations	91 456	36 393	80 515	75 881	101 433	147 778	533,456
Diagnostic category							
Influenza	14%	14%	24%	21%	28%	31%	24%
Pneumonia	68%	70%	60%	64%	57%	56%	61%
Bronchitis	17%	16%	16%	14%	15%	13%	15%
Proportion of SARI hospitalizations in ≥ 75 y age-group	53%	49%	55%	46%	60%	51%	53%
Number of hospitalizations with ICU admission	5 235	2 291	5 317	4 962	6 362	10 412	34,579
Proportion of hospitalizations with ICU admission	6%	6%	7%	7%	6%	7%	6%
Number of deaths	5 745	2 111	5 220	4 202	6 773	8 965	33,016
Proportion of deaths	6%	6%	6%	6%	7%	6%	6%

^a^Source: Santé publique France

### Estimated influenza-attributable SARI hospitalization rates

Hospitalization rates varied seasonally in both the periodic regression (14 to 108/100,000 inhabitants) and GLM (17 to 110 /100,000) (Table 2) methods. The 95% confidence intervals for the two methods overlapped for three influenza epidemics (2013–2014, 2016–2017, and 2017–2018) and were very similar for the 2015–2016 epidemic.

Estimated hospitalization rates using both methods were 1.8 times higher than hospitalization rates with a diagnosis of influenza alone (38/100,000).

**Table 2:** Estimated influenza-attributable SARI hospitalizations using periodic regression and GLM, per influenza epidemics, 2012–2018, metropolitan France

Influenza epidemic (Dominant influenza viruses*)	Periodic regression			GLM			Influenza***
	H	H/100,000	95% CI	H	H/100,000	95% CI	H/100,000
2012–2013 (B-Yamagata)	32 790	50.9	[44.3–57.4]	NA	NA	NA	21.5
2013–2014 (A(H1N1) et A(H3N2))	8 964	13.9	[11.1–16.7]	10 964	17.0	[15.1–18.9]	12.7
2014–2015 (A(H3N2))	38 519	59.7	[55.4–64.1]	44 804	69.5	[66.3–72.7]	34.8
2015–2016 (B-Victoria)	24 921	38.7	[34.2–43.1]	29 430	45.6	[43.7–47.6]	27.7
2016–2017 (A(H3N2))	52 354	81.2	[75.4–87.0]	49 817	77.3	[73.7–80.9]	45.9
2017–2018 (A(H1N1) et B-Yamagata)	69 610	108.0	[99.9–116.0]	70 780	109.8	[106.2-113.3]	69.0
Average of five epidemics**	**38 873**	**60.3**	**NC**	**41 159**	**63.8**	**NC**	**38.0**

*Source CNR of influenza viruses **influenza epidemics 2013–2014 to 2017–2018; H = hospitalizations; NA = not available; *** with a principal, related or associated influenza diagnosis, results from previous studies [2, 10]; NC = not calculated

### Influenza-attributable SARI hospitalization estimates according to diagnostic category, age-group and region of hospitalization (periodic regression)

Across the six epidemics, 227,154 influenza-attributable SARI hospitalizations were estimated, representing 43% of the 533,456 SARI hospitalizations (Table 3). During the epidemics, the proportion of influenza-attributable hospitalizations was estimated at 23% in pneumonia hospitalizations and 32% in those for bronchitis. The proportion of influenza-attributable decreased with age from 62% in the ≥ 85 years age group to 36% in the < 15 years age group (Table 3).

Over the six epidemics, the mean rate of hospitalizations varied significantly by age group: 23/100,000 inhabitants in those 15–64 years old to 473/100,000 in those ages ≥ 85 years old (Table 3). The mean crude hospitalization rate also varied by region: from 40.7/100,000 in Corsica to 73.4/100,000 in Provence-Alpes-Côte-D’azur (PACA). Overall, the average hospitalization rates observed in the eastern regions, and in particular in the southeast, were higher than in western regions, irrespective of the epidemic season (Additional file 3).

**Fig. 2:**
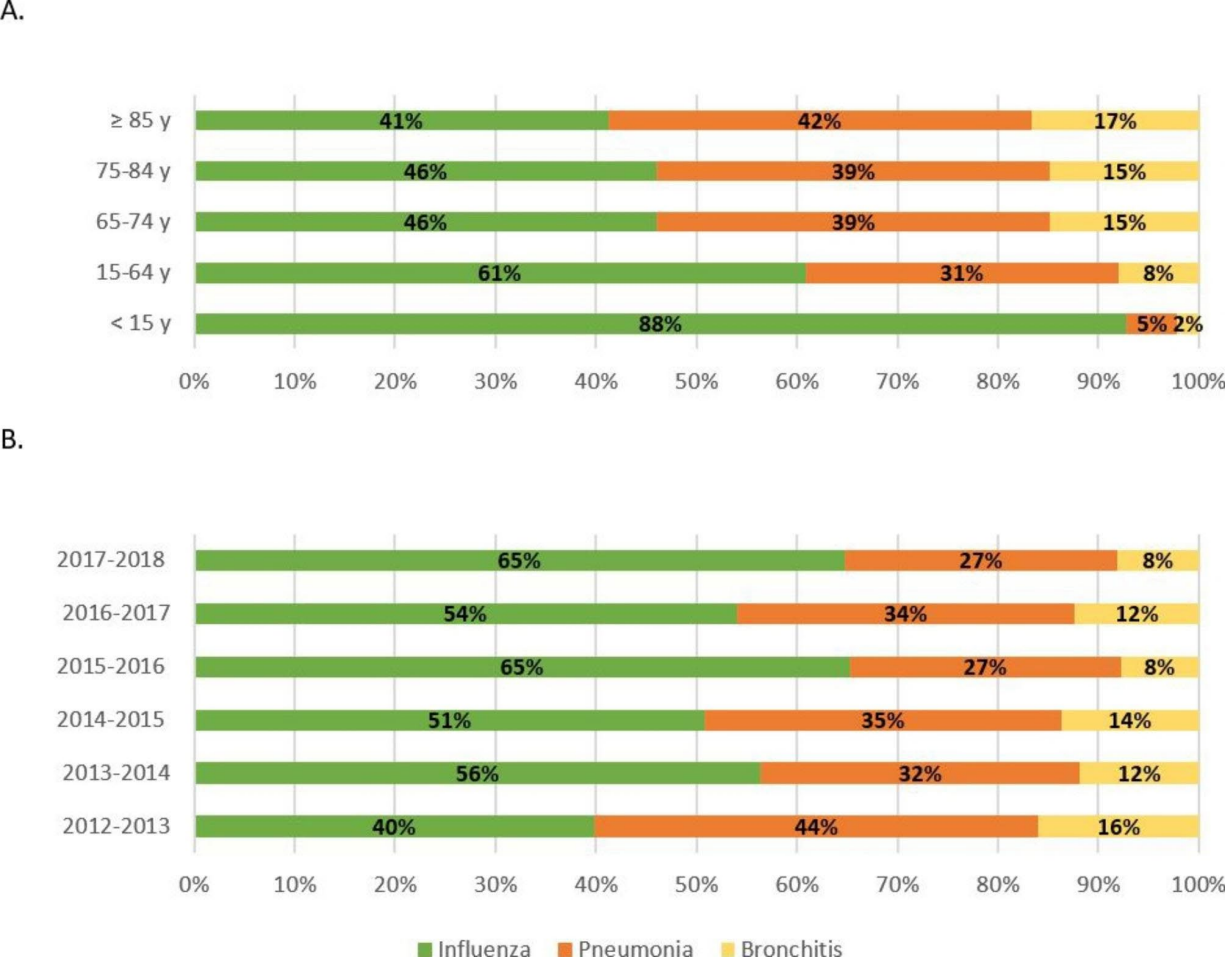
Distribution of estimated influenza-attributable SARI per diagnostic category, age group (A) and influenza epidemics (B), 2012–2018, metropolitan France

The distribution of the three diagnostic categories varied by age group, with the proportion of influenza diagnoses being twice as high in persons < 15 years old (88%) than those aged ≥ 85 years old (41%) (Fig. 2). The proportion of hospitalizations with influenza diagnosis also increased over time from 40 to 65% between the 2012–2013 and 2017–2018 epidemics.

**Table 3:** Distribution of estimated influenza-attributable SARI hospitalizations, stratified by diagnostic category and age group, 2012–2018, metropolitan France

	Number of SARI hospitalizations	Number of influenza-attributable SARI hospitalizations	Proportion of influenza-attributable SARI hospitalizations	SARI hospitalization rate (/100 000)
**Diagnostic category**				
Influenza	127 297	127 297	100	33
Pneumonia	326 062	74 223	23	19
Bronchitis	80 097	25 634	32	7
**Age group**				
< 15 y	60 104	36 308	60	52
15–64 y	119 746	56 742	47	23
65–74 y	71 427	29 452	41	78
75–84 y	122 338	48 043	39	200
≥ 85 y	159 841	56 917	36	473
**Total**	533 456	227 154	43	59

## Discussion

The analysis of SARI hospitalization data from the PMSI database allowed us to provide a more representative estimate of the true burden of influenza on France’s hospital system, as it took into account the non-visible part of influenza. Using periodic regression, we estimated that between the 2013–2014 and 2017–2018 influenza epidemics, 60/100,000 inhabitants were hospitalized per season. This estimate was confirmed by GLM, which provided a similar hospitalization rate of 64/100,000. In contrast, the hospitalization rate of patients with diagnosis of influenza alone was only two-thirds of the rate for influenza-attributable SARI. A previous study estimated the influenza hospitalization rate in metropolitan France at 38/100,000 per season over the period 2013 to 2018 [2, 10].

International comparisons are complicated by the diversity of definitions, sources, methods and study periods, as highlighted in the literature review by Roguski et, al. [11]. Based on estimates calculated from regression methods, the authors of that review estimated an average annual hospitalization rate of 57.8 / 100,000 inhabitants (95% CI: 51.6, 63.9 /100,000) for the period 2007 to 2018, which was very close to our estimates.

Our case definition of SARI was guided by recently published foreign experiences and by the WHO recommendation to develop surveillance of SARI to better assess the burden of influenza. This choice was based on the following arguments:

- Adopt a coherent syndromic grouping logic around respiratory infections and compatible with the WHO definition of SARI (acute low respiratory infection with a history of fever or measured fever ≥ 38 °C associated with a cough, occurring within the previous 10 days and requiring hospitalization) [5].
- Include in the definition of SARI: all influenza infections, whether coded as PD or AD (most hospitalizations with influenza as an AD were associated with a diagnosis it was reported as a complication or co-infection, and excess hospitalizations of respiratory infections (acute bronchitis and pneumonia) in order to compensate for the lack of confirmation of virological diagnosis of influenza [4, 12].
- Ensure consistency and comparability with choices made in other countries, most often ‘influenza and pneumonia’ with or without acute bronchitis [13–15], and more recently SARI [16, 17].

We chose to exclude hospitalizations where pneumonia or bronchitis was an AD with no mention of influenza. In the PMSI coding rules, the PD corresponds to the reason for admission and is therefore closer to the WHO definition of SARI.

The initial purpose of our study, conducted before the COVID-19 pandemic, was to ensure simplicity of implementation for regional and facility-level use. Rather than a complex model with unavailable co-variables at the regional or sub-regional geographic level, we chose a simple periodic regression method to define a ‘baseline’ of hospitalizations for pneumonia and bronchitis unrelated to influenza epidemics. The periodic regression model was therefore constructed by trimming the epidemic periods of influenza and bronchiolitis (proxy for RSV circulation) and limiting the estimated numbers of influenza-attributable cases to the epidemic period of influenza defined by the current surveillance system. Although our estimates were limited to the influenza epidemic periods, we were not able to fully take into account the circulation of other respiratory viruses, in particular RSV, which may be concomitant with influenza at the beginning of an epidemic. Multivariate studies including co-variables such as the positivity rate for winter viruses allow the overlap between RSV and influenza to be better taken into account. The GLM method we applied was adjusted for RSV circulation and provided seasonal estimates of the same order of magnitude as the periodic regression method, including seasons with concurrent circulation of influenza and RSV (2016-17 and 2017-18). This would suggest that the latter method was impacted very little by this limitation.

A previous study of hospitalizations with influenza diagnoses showed that the incidence, severity, lethality, and frequency of ICU hospitalizations in an epidemic season were all related to variations in the age distribution of cases during the epidemic rather than the virulence of the circulating subtype [18]. Our study shows an amplified effect of these variations in age-specific incidence with increasing age. The distinction between the three diagnostic categories comprising the syndromic grouping of SARI allowed us to objectify the important differences in the reasons for hospitalization according to agegroup. In people aged 65 years and over, the influenza-attributable SARI hospitalization rate was 2.2 times higher than the hospitalization rate including only influenza diagnosis (D09-J11), a difference similar to that observed by Reed et al. [14] in a study measuring the probability of being tested for influenza according to age. Over the six seasons between 2012 and 2018, the proportion of hospitalizations with influenza diagnosis increased from 40 to 65%, reflecting improved diagnostic testing practices for influenza, especially among adults and the elderly.

The periodic regression method used in this study made it possible to estimate the impact of influenza at the regional level and to confirm the existence of regional disparities in the use of hospitalizations for influenza across metropolitan France. This was previously observed in an earlier study on influenza [18] and more recently during the COVID-19 pandemic [19, 20]. It confirms that monitoring data on a sub-regional scale is essential to quantify the burden of seasonal epidemics, including influenza, and to adapt the care offer locally with a view to improving patient management.

Our study has the usual limitations associated with using medico-administrative databases whose coding rules are based on economic and not epidemiological criteria. We assumed that the coding of influenza diagnosis corresponded to virological confirmation. However, unlike other studies, we had no data to prove this [21, 22]. A previous French study on patients admitted to an ICU for influenza showed a good specificity of influenza coding in the PMSI database [23].

## Conclusion

The present study’s analysis of the PMSI medico-administrative database helped provide a more accurate estimation of the burden of influenza-associated SARI on France’s hospital system, particularly in the elderly. Moreover, this work complements an initial study that was limited to hospitalizations with a diagnosis of influenza, its objective was to describe characteristics and severity [18]. Both studies contribute to the overall evaluation of the socioeconomic impact of influenza in France. Their findings provide us with a better understanding of the burden of influenza on the hospital system, and should help raise the general public and health professionals’ awareness of the importance of barrier measures and influenza vaccination.

We only focused on hospitalizations in this study; other studies investigated overall (i.e., hospital and non-hospital) influenza-associated SARI mortality [3, 24]; their findings could contribute to provide an overall estimate of the burden.

This study covered a period prior to the SARS-Cov-2 pandemic that had a major impact on hospital activity. For comparison, the first wave of COVID-19 in France (March to May 2020) resulted in a hospitalization rate of 140/100,000 [25], higher than the 110/100,000 estimated during the large A(H3N2) influenza outbreak in 2017–2018.

The SARS-Cov-2 pandemic has led to a change in the dynamics of winter respiratory epidemics. For the next five years at least, the methods used to estimate hospitalizations based on the observation of previous periods must be reconsidered. Two main factors will have to be taken into account: viral interference between the three major respiratory viruses (influenza virus, SARS-Cov-2, and RSV) and the evolution of diagnostic confirmation practices given clinical pictures of SARI in order to adapt care management modalities [26, 27]. Consequently, the periodic regression method based on learning from previous observations will not be very effective for estimating excess hospitalizations for SARI attributable to the different viruses. The construction of a GLM using proxies adapted to the different viruses should be the preferred choice.

Finally, implementing prevention measures (e.g., face masks) against one respiratory virus lowers the aggregated risk of all viral respiratory infections. Thresholds of overall incidence of acute respiratory infections could therefore constitute simplified indicators for the recommendation of these prevention measures [28].

## Electronic supplementary material

Below is the link to the electronic supplementary material.

Additional file 1

Additional file 2

Additional file 3

## Data Availability

The datasets used and/or analyzed during the current study are not publicly available for legal and ethical reasons. The datasets are available from the corresponding author on reasonable request and only in compliance with the legal provisions allowing access to France’s most comprehensive health database, the National Health Data System (Système national des données de santé). More specifically,approval from the CESREES (Comité éthique et scientifique pour les recherches, les études et les évaluations dans le domaine de la santé) ethics committee and from the French data protection authority is needed.

## Abbreviations

AD: Associated diagnosis
AIC: Akaike Information Criterion
ED: Emergency departments
GLM: Generalized linear model
ICU: Intensive care units
ILI: Influenza-like illness
PACA: Provence-Alpes-Côte-D’azur
PD: Principal diagnosis
PMSI: French national hospital discharge database
RSV: Respiratory syncytial virus
SARI: Severe acute respiratory infections
SNDS: France’s most comprehensive National Health Data System (Système national des données de santé)
WHO: World Health Organization
