# Supplementary material for: Estimating the burden of influenza-attributable severe acute respiratory infections on the hospital system in Metropolitan France, 2012–2018
Source: BMC Infect Dis. 2023 Mar 6;23:128. doi: 10.1186/s12879-023-08078-2 (PMC9987108; doi:10.1186/s12879-023-08078-2)
Supplement: Supplementary file 1 — Additional file 1 [file 12879_2023_8078_MOESM1_ESM.pdf]

Additional file 1: Epidemic seasons of influenza and bronchiolitis

Supplementary box 1. Distribution of epidemic seasons of influenza and bronchiolitis per week, 2012-2018, metropolitan France

			Nov					Dec				Jan				Feb					Mar				Apr		
**2012-2013**	43	44	45	46	47	48	49	50	51	52	1	2	3	4	5	6	7	8	9	10	11	12	13	14	15	16	17
Influenza																											
Bronchiolitis																											
**2013-2014**	43	44	45	46	47	48	49	50	51	52	1	2	3	4	5	6	7	8	9	10	11	12	13	14	15	16	17
Influenza																											
Bronchiolitis																											
**2014-2015**	43	44	45	46	47	48	49	50	51	52	1	2	3	4	5	6	7	8	9	10	11	12	13	14	15	16	17
Influenza																											
Bronchiolitis																											
**2015-2016**	43	44	45	46	47	48	49	50	51	52	1	2	3	4	5	6	7	8	9	10	11	12	13	14	15	16	17
Influenza																											
Bronchiolitis																											
**2016-2017**	43	44	45	46	47	48	49	50	51	52	1	2	3	4	5	6	7	8	9	10	11	12	13	14	15	16	17
Influenza																											
Bronchiolitis																											
**2017-2018**	43	44	45	46	47	48	49	50	51	52	1	2	3	4	5	6	7	8	9	10	11	12	13	14	15	16	17
Influenza																											
Bronchiolitis																											
