# Supplementary material for: Estimating the burden of influenza-attributable severe acute respiratory infections on the hospital system in Metropolitan France, 2012–2018
Source: BMC Infect Dis. 2023 Mar 6;23:128. doi: 10.1186/s12879-023-08078-2 (PMC9987108; doi:10.1186/s12879-023-08078-2)
Supplement: Supplementary file 2 — Additional file 2 [file 12879_2023_8078_MOESM2_ESM.pdf]

Additional file 2: Generalized linear model parameters

Supplementary Fig 1. Weekly influenza-like illness (ILI) incidence data, 2013-2018, metropolitan France (source: French general practitioner network Sentinelles)

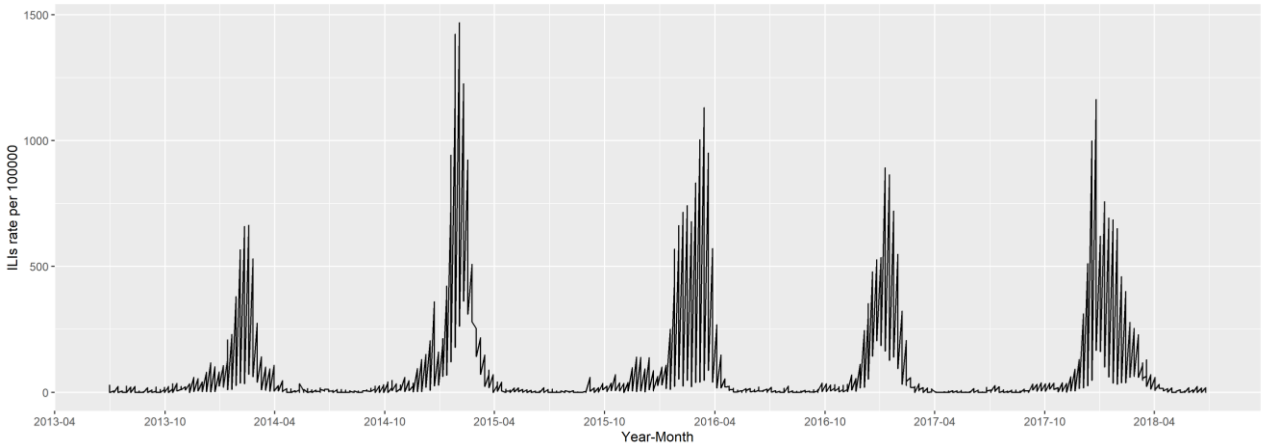

Data available at: https://www.sentiweb.fr/france/fr/?page=table

Supplementary Fig 2. Distribution of nasopharyngeal samples testing positive for influenza by influenza type and subtype: A(H1N1)pdm09, A(H3N2) and B, and by epidemics seasons, 2013-2018, metropolitan France (source : National Reference Center for Influenza)

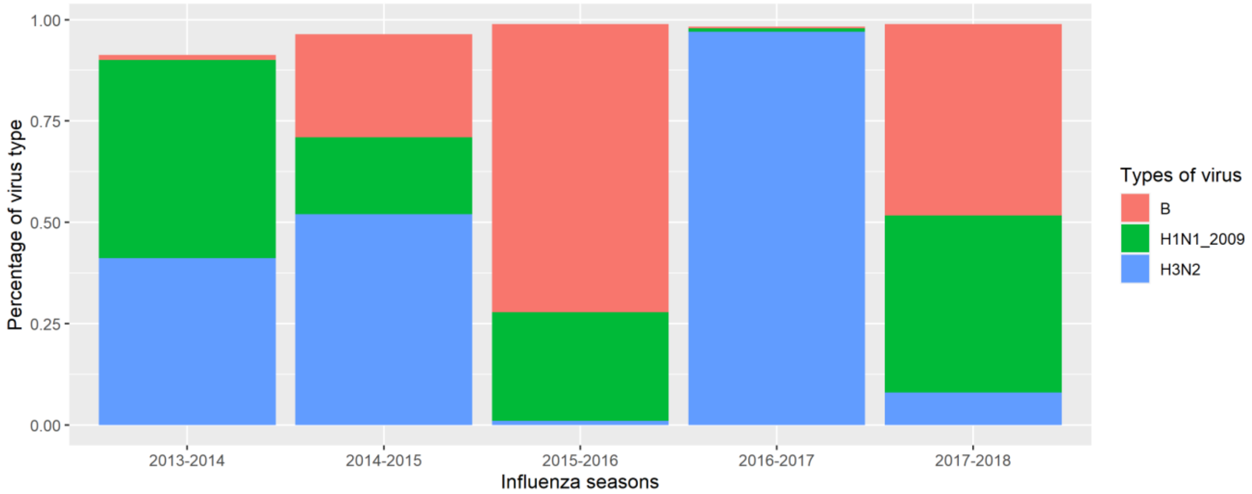

Data available in the activity reports of the National Reference Centre for Influenza: https://www.pasteur.fr/fr/sante-publique/centres-nationaux-reference/cnr/virus-infections-respiratoires-dont-grippe/rapports-activite-du-cnr-virus-infections-respiratoires-dont-grippe

Supplementary Fig 3. Distribution of indicators of influenza activity (for each influenza type and subtype this was the product of ILI incidence and the percentage of samples testing), 2013-2018, metropolitan France

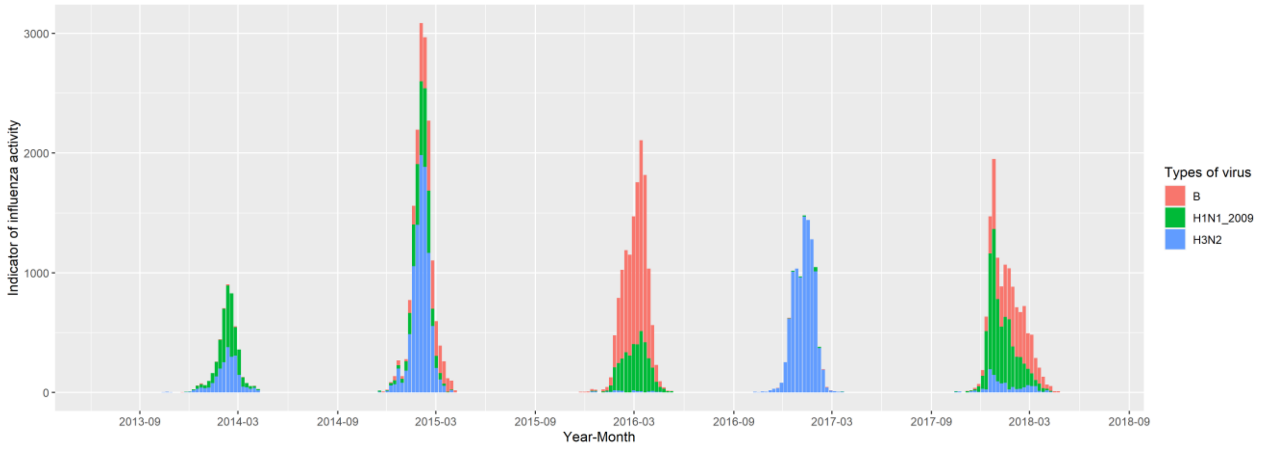

Supplementary Fig 4. Weekly proportions of consultations for bronchiolitis, 2013-2018, metropolitan France (source: emergency departments participating in the OSCOUR® network)

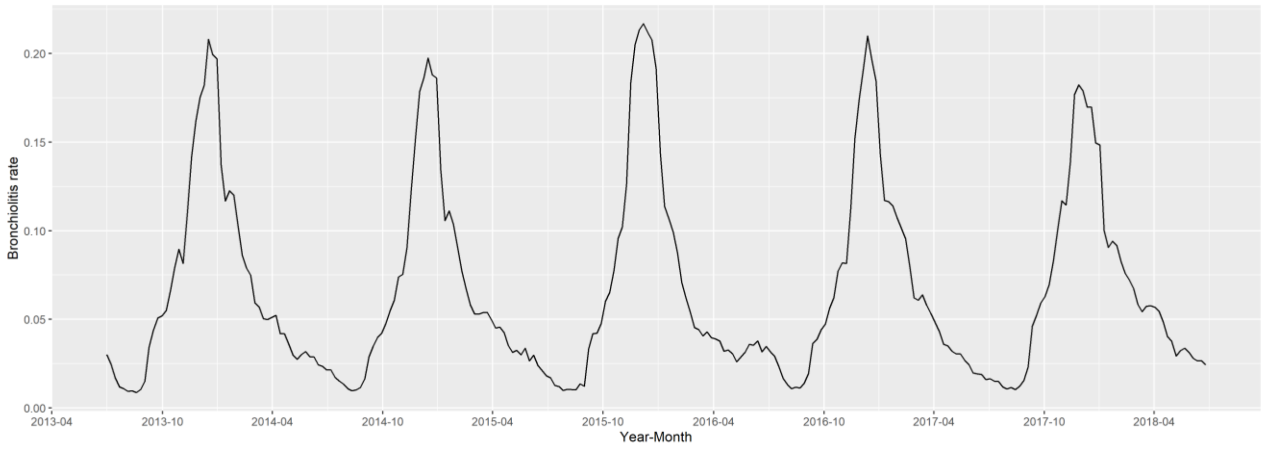

Data available at: https://geodes.santepubliquefrance.fr/#c=home
