# Supplementary figures and images for: Estimating the burden of influenza-attributable severe acute respiratory infections on the hospital system in Metropolitan France, 2012–2018
Source: BMC Infect Dis. 2023 Mar 6;23:128. doi: 10.1186/s12879-023-08078-2 (PMC9987108; doi:10.1186/s12879-023-08078-2)

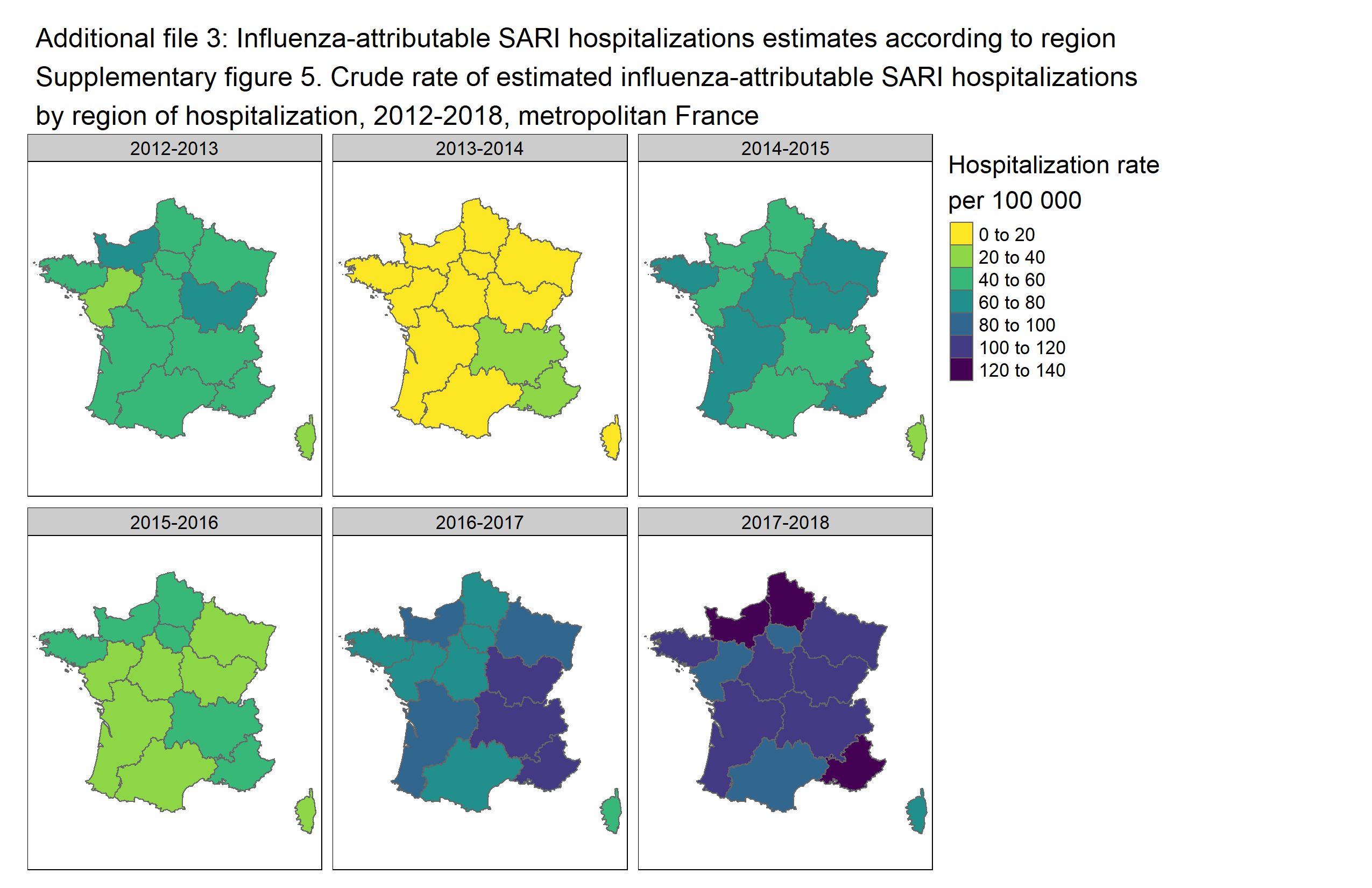

Supplement: Supplementary file 3 — Additional file 3 [file 12879_2023_8078_MOESM3_ESM.png]
